# Urinary Tract Abscess in Nephrectomized Renal Transplant Recipient

**DOI:** 10.4103/0974-777X.56253

**Published:** 2009

**Authors:** Reza Afshar, Siamak Afshin-Majd, Suzan Sanavi

**Affiliations:** *Departments of Nephrology and Neurology, Shahed University, Mustafa Khomeini Hospital, Italia St, Tehran, Iran*

Sir,

We write about an interesting case with unusual manifestations of urinary tract infection which misled physicians. A 40-year-old man presented with a 20-day history of severe left flank and low back pain radiating to the back of his left thigh with nausea and vomiting. He was a known case of end-stage renal disease who had undergone successful renal transplantation 12 years ago. Because of recurrent urinary tract infections he had been nephrectomized bilaterally six months pre transplant. No recent trauma and medical interventions had been reported.

He consumed 75 mg cyclosporine (in a low dose because of chronic allograft nephropathy), one gram mycophenolate mofetil, five mg prednisolone, and antihypertensive drugs. On physical examination, normal body temperature, pallor, left flank tenderness, diminished left side breath sounds and normal transplanted kidney were found. Because of low back pain radiating to lower limb associated with flank tenderness; absence of native kidneys, fever and urinary symptoms accompanied with normal transplanted kidney; the patient had been worked up by a neurologist for spinal nerve compression. Plain radiography of vertebral column showed extensive degenerative joint disease. However, neurologic exams were normal. If the patient had native kidneys, following diagnoses became propounded: nephrolithiasis, urinary tract obstruction and infection, lumbar disc disease and spondylosis, splenic and abdominal abscesses.

**Figure 1:**
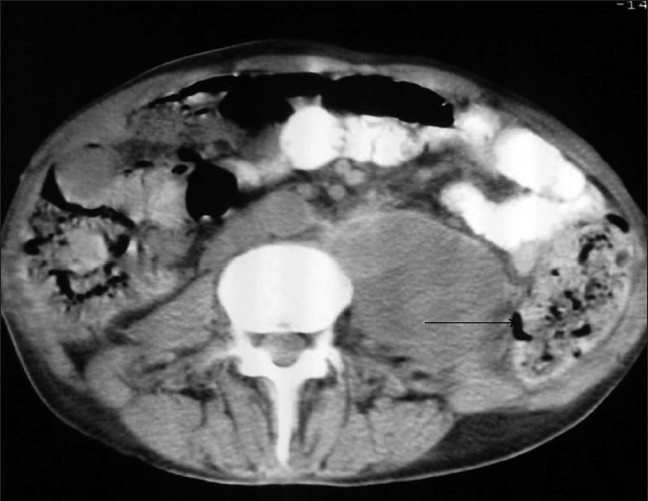
Spiral CT scan shows abscess

Laboratory investigations revealed elevated serum creatinine and erythrocyte sedimentation rate (ESR),; abnormal urine with many granular casts, protein and positive colony count for *pseudomonas aeroginosa*, susceptible only to piperacillin; and negativeblood culture. Ultrasonography showed a 75-mm heterogeneous cyst in previous left kidney site confirmed by spiral computed tomography (CT) scan [Figure 1, arrow]. The presence of such cysts in imaging studies is a typical feature of renal abscess. However, the patient had been nephrectomized and this abscess was accompanied with positive urine culture for *P. aeroginosa* which indicates left kidney remnant or retroperitoneal abscess penetration into native urinary tract. Incidence of infection is significantly higher in renal transplant recipients than general population. Although, among solid-organ transplants, kidney transplantation is associated with the lowest rates of infections, in part because of the elective or semielective nature of kidney transplantation These patients have multiple risk factors for infection including defect in immune system that result in decreased host resistance to infection.[1] Urinary tract infection is traditionally the most common bacterial infection occurring in the renal transplant recipient, particularly in the first few months post transplant;[2,3]. After posttransplant month 6, patients generally can be categorized as those with successful graft outcome, those with poor graft function because of chronic rejection and those chronically infected with immunomodulating viruses such as cytomegalovirus. Infections in patients with long-term successful allografts are typically similar to those that develop in persons in the community, while the latter two patient groups are at ongoing risk of opportunistic infections.[2–5] However, bacterial infections are common in the late posttransplant period but native urinary tract infection occurs rarely, particularly in a nephrectomized patient and this may mislead the physician because of unavailability of native kidneys and their little role in urine production.[4] The patients' abscess was drained under CT-guidance which contained suppuration due to the same pathogen and anti-Pseudomonas antibiotic (piperacillin) was initiated. The patient responded to this combination therapy and subsequently showed relative improvement in renal function. It must be remembered that steroid administration subsides inflammation presentations in renal transplant recipients.Thus, infection may progress disease and the clinicians must consider this problem and examine their patients thoroughly.
